# Patient-reported continued benefits of pexidartinib for tenosynovial giant cell tumor based on a real-world study in the United States

**DOI:** 10.1093/oncolo/oyaf028

**Published:** 2025-03-31

**Authors:** Dong Dai, Irene Pan, Klaus Freivogel, Xin Ye, Kristen Tecson, William Tap

**Affiliations:** Daiichi Sankyo, Inc., Basking Ridge, NJ 07920, United States; United BioSource LLC, Blue Bell, PA 19422, United States; United BioSource LLC, Blue Bell, PA 19422, United States; Daiichi Sankyo, Inc., Basking Ridge, NJ 07920, United States; Daiichi Sankyo, Inc., Basking Ridge, NJ 07920, United States; Sarcoma Medical Oncology Service, Memorial Sloan Kettering Cancer Center, New York, NY 10065, United States

**Keywords:** pexidartinib, tenosynovial giant cell tumor, patient-reported outcome, symptom change

## Abstract

**Background:**

Pexidartinib (Turalio) is the only systemic therapy approved by the United States Food and Drug Administration for the treatment of patients with tenosynovial giant cell tumors (TGCT ) based on clinical benefits demonstrated in the Phase III ENLIVEN trial. The present study assessed longitudinal patient-reported outcomes of patients treated with pexidartinib for TGCT.

**Methods:**

A longitudinal study was conducted in adult patients who received pexidartinib via the Risk Evaluation and Mitigation Strategy in the United States. Two web-based surveys containing patient-reported outcome questionnaires were administered to eligible patients (at least 18 years of age, had taken at least 1 dose of pexidartinib before baseline survey and still on treatment with pexidartinib at the time of follow-up survey, had not participated in any clinical trials for pexidartinib, and could complete questionnaires in English). The first assessment was initiated in 2021 (baseline), and the second was completed in 2022-2023 (follow-up).

**Results:**

Of 83 eligible patients who completed baseline assessment, 45 were eligible for follow-up. Thirty-one of whom consented and 28 completed the assessment. At the time of the follow-up survey, the mean (range) treatment duration was 18.5 (10.6-36.2) months, mean (SD) age was 41.9 (13.70) years, and 67.7% of the patients were female. The most common tumor sites were in the knee (67.7%) and ankle (16.1%). At follow-up, over 85% of patients reported symptom improvement since initiation of treatment with pexidartinib, and nearly 70% of patients reported symptoms to be “Very much improved” or “Much improved.” From baseline to follow-up assessment, changes in patient-reported measures on physical function, pain, and stiffness were not statistically significant (*P* > .05).

**Conclusions:**

Findings from this longitudinal study showed continued benefit of pexidartinib in overall symptom improvement from patients’ perspectives after an additional year of continued use among patients with symptomatic TGCT.

**Implications for practice**

Pexidartinib is available to patients in the United States by managed distribution through the Turalio Risk Evaluation and Mitigation Strategy (REMS) program. In this web-based, longitudinal survey of adult patients in the Turalio REMS program, we analyzed the change in self-evaluated level of pain during longer term use of pexidartinib in the real-world settings. Our analysis revealed the sustained benefits over 12 months for patients who continued treatment with pexidartinib to manage symptomatic tenosynovial giant cell tumor.

## Introduction

Tenosynovial giant cell tumors (TGCTs) are rare, benign, but locally aggressive neoplasms, characterized by inflammation of the synovial lining of the joints and tendons, which may cause significant symptom burden in afflicted patients.^1,2^ Tenosynovial giant cell tumor affects people of all ages; however, it is most typically seen in working-age adults and can lead to debilitating physical disabilities.^3,4^ Common symptoms of TGCT are pain, stiffness, edema, and restricted range of motion; severity and extent of disease activity vary by intra- and extra-articular involvements of muscles, ligaments, and tendons.^4,5^

Pexidartinib (Turalio, Daiichi Sankyo, Inc.) is the only systemic therapy approved by the United States Food and Drug Administration for the treatment of adult patients with symptomatic TGCT associated with severe morbidity or functional limitations and not amenable to improvement with surgery.^6^ The approval was based on the double-blind, randomized, placebo-controlled Phase III ENLIVEN trial, which demonstrated a 39% overall response rate and meaningful improvements in physical function and stiffness with pexidartinib at week 25 in 120 patients with advanced TGCT.^7,8^ Because of the risk of a rare and idiopathic cholestatic hepatotoxicity, pexidartinib is available to patients who are registered in the Turalio Risk Evaluation and Mitigation Strategy (REMS) program in the United States.^6^ The Phase III ENLIVEN trial included an open-label study follow-up, and among 61 patients who started on pexidartinib from the randomized trial and continued to receive pexidartinib in the open-label study, the weekly mean worst pain numerical rating scale (NRS) score changed (from baseline) by −2.7 ± 2.2 after 25 weeks (33 of 61) and by −3.3 ± 1.7 after 50 weeks (22 of 61), showing that pain relief appeared to be sustained for at least 50 weeks.^9^

The benefit of pexidartinib was also assessed in real-world clinical settings and was first reported in a survey study of patients enrolled in the Turalio REMS program.^10^ The survey study included 83 patients and reported symptom improvements during pexidartinib treatment compared to the time prior to pexidartinib treatment. The objective of the current survey study was to follow up on patients who completed this previous survey study and remained on treatment with pexidartinib to collect patient-reported outcomes associated with continued use of pexidartinib beyond 12 months in real-world clinical settings.

## Methods

Two web-based surveys were administered between May 2021 and February 2023. The first survey (baseline assessment) was administered between May and July 2021 to patients enrolled in the Turalio REMS program who had initiated treatment with pexidartinib for TGCT. After which, a second survey (follow-up assessment) was collected between April 2022 to February 2023 to assess patients’ experiences with continuous use of pexidartinib. Study patients received an honorarium for completing the surveys. In addition to the data collected directly from patients through the web-based survey, clinical data collected at the time of registering patients in REMS were obtained from the REMS database to describe patients’ demographics and baseline clinical characteristics.

Results of the baseline assessment on 83 patients were previously published.^10^ The study protocol and follow-up assessment survey were reviewed and approved by Advarra Institutional Review Board on March 9, 2022.

### Survey population

Study patients were recruited from the Turalio REMS program. Patients who were at least 18 years of age, had taken at least 1 dose of pexidartinib, had not participated in any clinical trials for pexidartinib, and could complete questionnaires in English were eligible. Eighty-three patients completed the baseline assessment (28.8% of the 288 invited), including 22 who had already discontinued or were withholding treatment with pexidartinib at the time. Invitations for the follow-up assessment were sent to a subset (*n* = 45) of these 83 patients who were still on treatment with pexidartinib at the time of follow-up survey recruitment. To qualify, patients also could not be enrolled in any clinical trial at the time of completing the follow-up survey. Eligible patients who were interested in completing the survey were presented with an online informed consent form, and only those who consented proceeded to the online survey.

### Survey development

The web-based survey was programmed and hosted using QuestionPRO for the baseline assessment and ClinCapture for the follow-up assessment. Both electronic data collection tools are Health Insurance Portability and Accountability Act–compliant. Compared with the baseline assessment, the follow-up assessment was abridged and contained patient-reported outcome assessments only.

From the 121-item bank of Patient-Reported Outcomes Measurement Information System (PROMIS)—Physical Function (PF), a 13-item module was customized to assess mobility among patients with lower-extremity tumors, and an 11-item module was customized to assess upper-limb function among patients with tumors in the upper-extremity.^11^ PROMIS-PF scores are expressed as T-scores, where a higher score corresponds to better physical function, and a score of 50 represents the average level of physical functioning with a SD of 10 in the US general population.^12^

Stiffness and pain in the joints were evaluated using the 1-item Worst Stiffness NRS and 1-item Worst Pain NRS. The Worst Stiffness NRS assesses the worst stiffness ranging from 0 (no stiffness) to 10 (stiffness as bad as imaginable). The Worst Pain NRS assesses the worst pain ranging from 0 (no pain) to 10 (pain as bad as imaginable). At baseline assessment, patients were asked to recall perceived stiffness and pain in the affected joints for 3 periods: before initiating pexidartinib, on most days treated with pexidartinib, and on the day of baseline survey completion. At follow-up assessment, patients were asked to rate stiffness and pain at the time of follow-up assessment completion.

The 14-item Treatment Satisfaction Questionnaire for Medication (TSQM) was used to evaluate 3 domains of treatment satisfaction: effectiveness, side effects, and convenience.^13^ One item summarizes a patient’s overall impression with a global satisfaction rating. Each domain score and the one-item global satisfaction rating ranges between 0 and 100, with higher scores indicating higher satisfaction.

Lastly, patients were asked to assess their overall impression of change to-date since starting treatment with pexidartinib on a 7-point Patient Global Impression of Change (PGIC) scale.^14^

### Data analysis

A descriptive analysis was conducted to summarize the survey responses. Continuous data are presented as mean, SD, median, and range (minimum and maximum values). Categorical variables are presented as frequencies with percentages. Paired Student’s *t*-tests were performed for the Worst Stiffness and Worst Pain NRS across different recall time periods. A random slope regression model was developed to estimate the average rate of change in PROMIS-PF score over time since the first pexidartinib dose. The level of statistical significance was set at 0.05. Treatment duration was calculated as the period from the date of the first dose up to the date of completion of the follow-up assessment. Statistical analyses were performed using R version 4.3.3 (R Foundation for Statistical Computing) or SAS version 9.4 (SAS Institute).

## Results

### Patient characteristics

**Figure 1.**
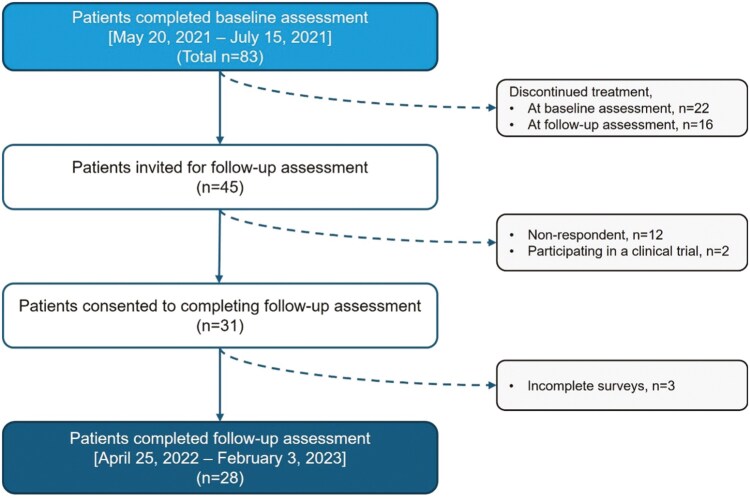
Patient attrition.

The follow-up assessment was fielded on April 3, 2022 and remained open until February 3, 2023. Among the 45 patients who were potentially eligible for the follow-up assessment, 2 were participating in clinical trials and 12 did not respond to the study invitation. Thirty-one patients consented, and 28 completed the follow-up assessment (Figure 1). The median (range) treatment duration was 7.6 (0.4-28.0) months at baseline and 19.6 (10.6-38.9) months at follow-up.

Patient demographic and clinical characteristics are summarized in Table 1. For the follow-up survey patients, the mean (SD) age at REMS registration was 41.9 (13.7) years, and this survey sample was predominantly female (67.7%) and White (67.7%), as was the baseline survey cohort. Geographic distribution was similar across 4 regions in the United States. Most patients had tumors in their lower extremities (29; 93.5%), with the knee (67.7%) and ankle (16.1%) being the joints most frequently affected (Table 1). One patient had multiple lesions in the ankle and foot, and another patient had lesions in the knee, hip, and ankle. The majority (90.3%) of patients had no existing hepatic comorbidities; one patient had diabetes, one had viral hepatitis, and one had biliary tract disease, gallbladder disease, and a family history of liver disease. The follow-up survey patients were a subset of the baseline survey patients (37.3%; 31/83). Overall, this subset is not significantly different from the baseline survey patients (Table 1).

**Table 1.** Demographic and clinical characteristics.

Variable	Baseline assessment patients (*n* = 83)^a^	Follow-up assessment patients (*N* = 31)^a^
Age,^b^ mean (SD), y	44.2 (14.1)	41.9 (13.7)
Sex, *n* (%)		
Female	52 (62.7)	21 (67.7)
Male	30 (36.1)	10 (32.3)
Missing	1 (1.2)	0 (0)
Race, *n* (%)		
White	54 (65.1)	21 (67.7)
Asian	6 (7.2)	5 (16.1)
Black or African American	7 (8.4)	3 (9.7)
American Indian or Alaskan Native	0 (0)	0 (0)
Native Hawaiian or Other Pacific Islander	0 (0)	0 (0)
Other	15 (18.1)	2 (6.5)
Missing	1 (1.2)	0 (0)
Geographic location, *n* (%)		
Midwest	22 (26.5)	9 (29.0)
Northeast	18 (21.7)	8 (25.8)
West	21 (25.3)	8 (25.8)
South	20 (24.1)	5 (16.1)
US Territories	1 (1.2)	1 (3.2)
Missing	1 (1.2)	0 (0)
Tumor location, *n* (%)		
Knee	51 (61.4)	21 (67.7)^c^
Hip	10 (12.0)	3 (9.7)^c^
Ankle	10 (12.0)	5 (16.1)^c^
Foot	7 (8.4)	3 (9.7)^c^
Wrist	3 (3.6)	1 (3.2)
Shoulder	2 (2.4)	1 (3.2)
Elbow	2 (2.4)	0 (0)
Hand/fingers	3 (3.6)	0 (0)
Hepatic medical history,^a^^,^^d^ *n* (%)		
No hepatic medical history	73.0 (88.0)	28 (90.3)
Hepatitis viral status	3 (3.6)	1 (3.2)
Family history of liver disease	2 (2.4)	1 (3.2)
Biliary tract disorder	1 (1.2)	1 (3.2)
Gallbladder disease	1 (1.2)	1 (3.2)
Diabetes	4 (4.8)	1 (3.2)
Hypertriglyceridemia	1 (1.2)	0 (0)

^a^Percentage was calculated based on 83 baseline assessment completers and 31 follow-up assessment completers, respectively.

^b^Data obtained from the Turalio REMS program patient enrollment forms.

^c^One patient had lesions in the ankle and foot, and one patient had lesions in the knee, hip, and ankle.

^d^One patient may have more than one existing hepatic comorbidity.

Abbreviations: *n*, number of patients; REMS, risk evaluation and mitigation strategy; y, year.

### Physical function

The mean (SD) standardized PROMIS-PF T-score was 43.34 (7.649) at follow-up assessment compared with 44.17 (8.028) at baseline assessment (Table 2). The score range was 32.8-61.4 for both baseline and follow-up assessments. From baseline to follow-up assessment, the mean (SD) change in PROMIS-PF score was –0.2 (1.218) and was not a statistically significant change (*P* = .3622) after adjustment for treatment duration of pexidartinib (Table 2).

**Table 2.** Patient-reported physical function.

Baseline PROMIS-PF T-score	
*n*	31
Mean (SD)	44.17 (8.028)
Median (range)	42.7 (32.8-61.4)
**Follow-up PROMIS-PF T-score**	
*n*	28
Mean (SD)	43.34 (7.649)
Median (range)	41.7 (32.8-61.4)
**Change in PROMIS-PF T-score from baseline**	
*n*	28
Mean (SD)	−0.2 (1.218)
Median (range)	0 (−4.3 to 1.5)
*P*-value	.3622

Abbreviations: *n*, number of patients; PROMIS-PF, patient-reported outcome measurement information system—physical function.

### Pain and stiffness

**Figure 2.**
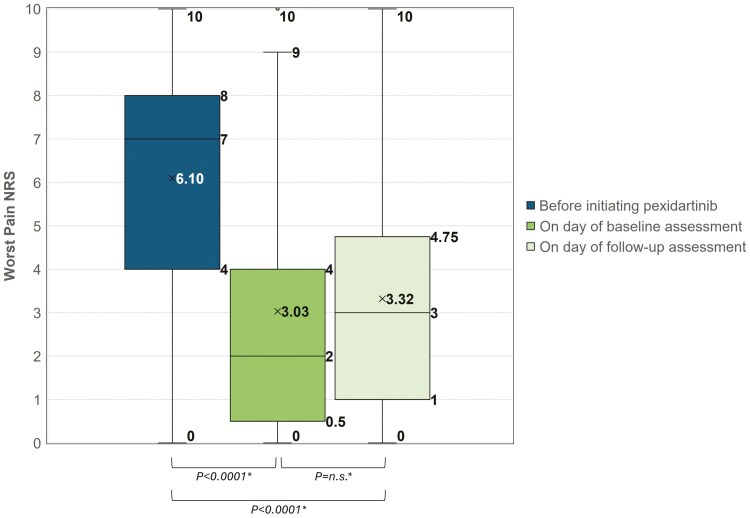
Worst pain NRS before treatment with pexidartinib recalled at baseline (*n* = 31) and rated at baseline (*n* = 31) and follow-up (*n* = 28). “x” marks the mean value of Worst Pain NRS. *Paired *t*-test of the mean. NRS, numeric rating scale; n.s., not statistically significant.

**Figure 3.**
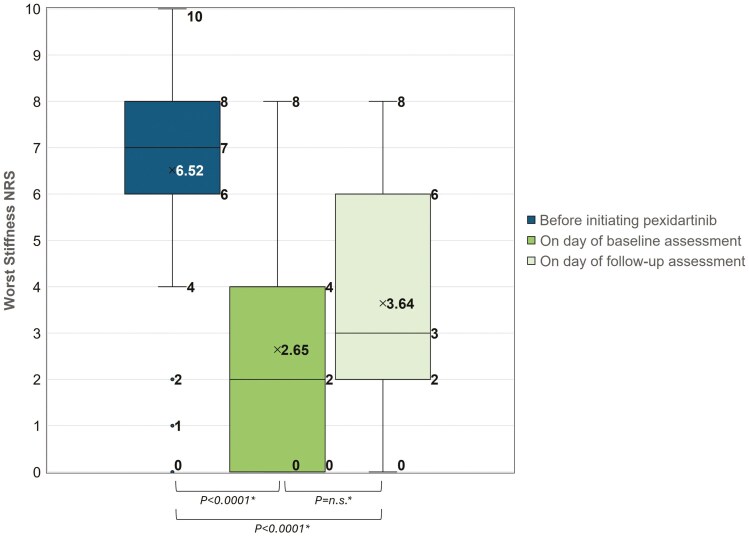
Worst stiffness NRS before treatment with pexidartinib recalled at baseline (*n* = 31) and rated at baseline (*n* = 31) and follow-up (*n* = 28). “x” marks the mean value of Worst Stiffness NRS. *Paired *t*-test of the mean. NRS, numeric rating scale; n.s., not statistically significant.

Results on Worst Pain NRS and Worst Stiffness NRS are summarized in Table 3. Figures 2 and 3 present the boxplots of scores for the 3 recall time points: before initiating pexidartinib, on the day of baseline assessment, and on the day of follow-up assessment. The mean (SD) Worst Pain NRS at baseline was 3.03 (2.89), which is a 3.06-point reduction (*P* < .0001) from 6.10 (2.88) before taking pexidartinib (Table 3). At baseline assessment, 71.0% (22/31) patients reported Worst Pain NRS reduction that exceeded the clinically meaningful threshold of ≥ 2 point change.^15^ At follow-up assessment, the mean (SD) Worst Pain NRS was 3.32 (2.78) compared to 3.03 (2.89) at baseline assessment (*P* = n.s.) (Table 3).

**Table 3.** Patient-reported worse stiffness and worst pain numeric rating scale scores by recall time periods.

	(A) Before initiating pexidartinib	(B) On the day of baseline assessment^a^	(C) On the day of follow-up assessment^b^
**Worst stiffness NRS**			
*n*	31	31	28
Mean (SD)	6.52 (2.77)	2.65 (2.61)	3.64 (2.45)
Median (range)	7 (0-10)	2 (0-8)	3 (0-8)
Reduction on NRS ≥ 1, *n* (%)	-	23 (74.2)^c^	25 (89.3)^d^
Comparing to (A) before initiating pexidartinib:			
Change in NRS, mean (SD)	-	−3.87 (3.14)^c^	−3.25 (2.81)^d^
*P*-value^e^	-	<.0001^c^	<.0001^d^
Comparing to (B) on the day of baseline assessment:			
Change in NRS, mean (SD)	-	-	0.75 (2.72)^f^
*P*-value^e^	-	-	n.s.^f^
**Worst pain NRS**			
*n*	31	31	28
Mean (SD)	6.10 (2.87)	3.03 (2.89)	3.32 (2.77)
Median (range)	7 (0-10)	2 (0-10)	3 (0-8)
Reduction on NRS ≥ 2, *n* (%)	-	22 (71.0)^c^	20 (71.4)^d^
Comparing to (A) before initiating pexidartinib:			
Change in NRS, mean (SD)	-	−3.06 (2.90)^c^	−3.14 (2.72)^d^
*P*-value^e^	-	<.0001^c^	<.0001^d^
Comparing to (B) on the day of baseline assessment:			
Change in NRS, mean (SD)	-	-	0 (2.09)^f^
*P*-value^e^	-	-	n.s.^f^

^a^Baseline assessment: first survey after initiating pexidartinib.

^b^Follow-up assessment: second survey after initiating pexidartinib.

^c^Comparing (B) vs (A).

^d^Comparing (C) vs (A).

^e^Statistical significance was determined using paired Student’s *t*-test.

^f^Comparing (C) vs (B).

Abbreviations: *n*, number of patients; n/a, not applicable; n.s., not statistically significant; NRS, numeric rating scale; PROMIS-PF, Patient-Reported Outcomes Measurement Information System—physical function.

The mean (SD) Worst Stiffness NRS at baseline was 2.65 (2.61), which is a 3.87-point reduction (*P* < .0001) from 6.52 (2.77) before taking pexidartinib, and 74.2% (23/31) patients reported Worst Stiffness NRS reduction that exceeded the clinically meaningful threshold of ≥ 1 points change.^16^ On the day of follow-up assessment, the mean (SD) Worst Stiffness NRS was 3.64 (2.45) compared to 2.65 (2.61) on the day of baseline assessment (*P* = n.s.) (Table 3).

When compared to that before taking pexidartinib, both the Worst Pain NRS and Worst Stiffness NRS on the day of follow-up were still showing significant reduction (*P* < .0001) (Table 3).

### Treatment satisfaction and global impression of change

The mean (SD) TSQM global treatment satisfaction was 70.2 (18.753). Across the 3 domains of TSQM, effectiveness was rated with the highest satisfaction (74.0 [18.021]) compared with the side effects (59.8 [22.658]) and convenience (63.5 [19.211]) domains (Table 4).

**Table 4.** Treatment satisfaction and global impression of change in disease status since initiation of pexidartinib.

Variable	Follow-up assessment (*n* = 28)
TSQM global satisfaction domain score	
Mean (SD)	70.2 (18.753)
Median (range)	71.4 (35.7-100.0)
TSQM effectiveness domain score	
Mean (SD)	74.0 (18.021)
Median (range)	75.0 (38.9-100.0)
TSQM side effects domain score	
Mean (SD)	59.8 (22.658)
Median (range)	56.3 (12.5-100.0)
TSQM convenience domain score	
Mean (SD)	63.5 (19.211)
Median (range)	63.9 (27.8-100.0)
PGIC on overall condition since initiating pexidartinib, *n* (%)	
Very much improved	4 (14.3)
Much improved	15 (53.6)
Minimally improved	5 (17.9)
No change	4 (14.3)
Minimally worse	0
Much worse	0
Very much worse	0

Abbreviations: PGIC, Patient Global Impression of Change; TSQM, treatment satisfaction questionnaire for medication.

Of the 28 patients who completed the follow-up assessment, the majority (24/28; 85.7%) reported improvement in overall joint symptoms: 14.3% (4/28) reported “Very much improved,” 53.6% (15/28) reported “Much improved,” and 17.9% (5/28) reported “Minimally improved.” The remaining 4 patients (14.3%) reported “No change.” No patient reported worsening in overall symptoms since initiation of pexidartinib (Table 4).

## Discussion

The objective of this study was to collect follow-up data on patients who completed a previous survey study (baseline) on treatment experience with pexidartinib and continued using pexidartinib since. The median (range) treatment duration was 18.5 (10.6-36.2) months at the time of follow-up assessment. The mean (SD) of PROMIS-PF was 44.17 (8.028) and 43.34 (7.649) at baseline and follow-up, respectively, which was within −1 SD from the mean T-score of 50 for the general population.^12^ This finding suggests that, on average, patients who continued pexidartinib treatment reported physical function at a level that was close to the general population. From baseline to follow-up, the change in mean (SD) of PROMIS-PF T-score was −0.2 (1.218), which was not a significant change either clinically or statistically. The median and range of PROMIS T-scores at baseline and follow-up assessments are also similar. This suggests a stability in physical functioning that was maintained over the approximate 12 months between baseline and follow-up assessments while patients continued treatment with pexidartinib with an accumulated treatment duration of approximately 11 to 36 months. Reports of the baseline and follow-up assessments of Worst Stiffness NRS and Worst Pain NRS take a similar trend, that the improvements are sustained between baseline and follow-up, with a mean (SD) change in Worst Stiffness NRS of 0.75 (2.72) and a mean (SD) change in Worst Pain NRS of 0 (2.09), and neither of the changes was statistically nor clinically significant.

The follow-up survey included only PRO instruments and no additional questions on treatment patterns; therefore, there could have been treatment interruptions amongst the 31 follow-up assessment patients who persisted on using pexidartinib that were not captured. Nevertheless, the majority of the 28 patients who completed the follow-up assessments showed sustained effectiveness in terms of physical functioning, pain, and stiffness and reported to be moderately satisfied with the treatment. At the time of follow-up assessment, 67.9% of the patients reported “Much improved” or “Very much improved” in overall condition since initiating pexidartinib on the PGIC; however, 5 patients (17.9%) reported “Minimally improved” and 4 patients (14.3%) reported “No change.” This is consistent with patients’ evaluation of treatment satisfaction by TSQM, where the mean domain score was highest with the effectiveness domain (74.0), followed by the convenience (63.5) and the side effects (59.8) domains. Of the 83 patients who completed baseline assessment, 22 had already discontinued or were withholding pexidartinib at the time of baseline assessment. Another 16 patients had discontinued pexidartinib sometime between the baseline and follow-up assessments. This finding suggests that effectiveness remains the most important driver of treatment persistency, while in the context of treatment tolerance, convenience and side effects are domains where improvements would further enhance patients’ overall treatment experience.

### Limitations

There are several limitations to consider when interpreting the results of the follow-up survey. One inherent to the direct-to-patient survey methodology is that patients are the only data source. In this case, objective assessment of treatment outcomes, such as radiological findings, was not available to evaluate patient-reported data on pain and stiffness in relation to tumor responses. Also, the baseline survey was subject to recall bias due to patients having to recall past symptoms and dosing information. As the follow-up assessment was designed to obtain outcomes data with reduced recall burden, the survey did not collect dosing information, and the analysis was not able to evaluate the correlation between dose and the reported outcomes. Furthermore, the follow-up cohort analyzed is a smaller sample of 28 patients of the original 83 patients. Attrition was mainly due to treatment discontinuation; hence, these results are subject to selection bias. Based on the results of the baseline assessment, it seems common for patients to discontinue as a precaution due to abnormal laboratory results for hepatic toxicity or experiencing side effects including hair color change and fatigue.^10^ As the follow-up survey was designed to assess outcomes associated with the use of pexidartinib beyond the 6-month treatment period in Phase III ENLIVEN trial, it did not include patients who discontinued treatment after completion of the baseline assessment. Therefore, these patients’ reasons for discontinuation were not available to provide additional insights on tolerability.

Overall, the follow-up cohort shares similar demographic and clinical characteristics with the original baseline cohort, which is comparable to the study populations from the ENLIVEN trial and the larger multi-national, multi-center TGCT Observational Platform Project disease registry as described previously.^10^ Therefore, the results based on the follow-up cohort can be interpreted as representative of the target patient population with continued use of pexidartinib in real-world clinical settings.

## Conclusions

The follow-up assessment provided additional real-world evidence on how patients benefitted from longer term use of pexidartinib for TGCT. At baseline assessment, most patients reported improvements in physical function, stiffness, pain, and overall symptoms by approximately 8 months after initiating pexidartinib (median duration of use 7.6 months). Results of the follow-up assessment showed that these clinical benefits were sustained among those who remained on pexidartinib for approximately another 10 to 12 months. The findings of this study are comparable to results from the ENLIVEN open-label study that reported sustained pain relief after week 50. Considering the availability of a 125 mg dose capsule of pexidartinib, further study is warranted to examine the impact of this new dosage on patient-reported outcomes.

## Data Availability

The data underlying this article are available in the article.
